# OLDER ADULT PET OWNERSHIP HISTORY, MARITAL STATUS, AND HEALTH OUTCOMES: THE WISCONSIN LONGITUDINAL STUDY (WLS)

**DOI:** 10.1093/geroni/igz038.725

**Published:** 2019-11-08

**Authors:** Alisha Hackney, Erika Friedmann

**Affiliations:** University of Maryland School of Nursing, Baltimore, Maryland, United States

## Abstract

We explore the association of PO and marital status to changes in health during aging. This secondary analysis uses 1992, 2004, and 2011 data from healthy participants (N=2,319) in the WLS. Health outcomes (symptoms, illnesses, days in bed due to illness, depression, and cognition) and demographic/ health variables (age, marital status, sex, smoking, BMI, engagement with friends/relatives). PO (ever owned a pet/dog/cat) was assessed in 2011. In linear mixed models, PO, dog ownership, and cat ownership (CO) independently predicted changes in health outcomes. Marital status moderated the association of CO to changes in cognition[F(1,012.85)=4.09,p=0.043]. Both marriage and CO were associated with increased cognition from ages 53 to 64; being unmarried without CO was associated with reduced cognition during aging with the lowest cognition by age 70. Results provide insight into how PO and social support influence healthy aging. Longitudinal PO and health/social data are necessary to better understand these relationships.

